# Efficacy and safety of once-daily fluticasone furoate 50 mcg in adults with persistent asthma: a 12-week randomized trial

**DOI:** 10.1186/s12931-014-0088-z

**Published:** 2014-08-11

**Authors:** Paul M O’Byrne, Ashley Woodcock, Eugene R Bleecker, Eric D Bateman, Jan Lötvall, Richard Forth, Hilary Medley, Loretta Jacques, William W Busse

**Affiliations:** Michael G DeGroote School of Medicine, Hamilton, ON Canada; Institute of Inflammation and Repair, University of Manchester, Manchester, UK; Center for Genomics and Personalized Medicine, Wake Forest University Health Sciences, Winston-Salem, NC USA; Department of Medicine, University of Cape Town, Cape Town, South Africa; Krefting Research Centre, University of Gothenburg, Gothenburg, Sweden; Quantitative Sciences Division, GlaxoSmithKline, Research Triangle Park, NC USA; Respiratory Medicine Development Centre, GlaxoSmithKline, London, UK; Department of Medicine, University of Wisconsin, Madison, WI USA

**Keywords:** Fluticasone furoate, Inhaled corticosteroid, Lung function, Once daily, Safety

## Abstract

**Background:**

Fluticasone furoate (FF) is a novel, once-daily inhaled corticosteroid (ICS) that has been shown to improve lung function vs. placebo in asthma patients. This study evaluated the efficacy and safety of FF 50 mcg compared with placebo in asthma patients uncontrolled by non-ICS therapy.

**Methods:**

This 12-week, multicentre, randomized, double-blind, placebo-controlled, parallel-group, phase III study randomized 248 patients (aged ≥12 years) to once-daily FF 50 mcg administered via the ELLIPTA™^a^ dry powder inhaler or placebo. The primary endpoint was change from baseline in pre-dose evening trough forced expiratory volume in one second (FEV_1_). Secondary endpoints were change from baseline in percentage of rescue-free 24-h periods (powered), evening and morning peak expiratory flow, symptom-free 24-h periods and withdrawals due to lack of efficacy. Other endpoints included Asthma Control Test™, Asthma Quality of Life Questionnaire and ELLIPTA ease of use questions. Safety was assessed throughout the study.

**Results:**

There was a significant difference in evening trough FEV_1_ between FF 50 mcg and placebo (treatment difference: 120 mL; *p =* 0.012). There was also a significant difference in rescue-free 24-h periods (11.6%; *p =* 0.004) vs. placebo. There were numerically greater improvements with FF vs. placebo for all remaining secondary endpoints. The incidence of adverse events was lower with FF (31%) than with placebo (38%); few were treatment-related (FF 50 mcg: n = 1, <1%; placebo: n = 4, 3%).

**Conclusion:**

FF 50 mcg once daily significantly improved FEV_1_ and percentage of rescue-free 24-h periods experienced over 12 weeks vs. placebo, and was well tolerated.

**Trial registration:**

www.clinicaltrials.gov, registration number: NCT01436071

## Background

Failure to achieve asthma control can impact patients’ daily lives and results in persistent symptoms, more frequent exacerbations and absenteeism from work and school [1,2]. Inhaled corticosteroids (ICS) are the most effective anti-inflammatory treatments for all severities of persistent asthma [3–5]. Patient adherence is a key component to the overall success of asthma treatment, and it has been demonstrated that compliance with a once-daily ICS is better than with a twice-daily regimen [6].

Fluticasone furoate (FF) is a novel once-daily ICS treatment for asthma [7–11], which is also used in combination with the long-acting β_2_-agonist (LABA) vilanterol (VI) for the once-daily treatment of asthma and COPD [12–14]. Animal and human pharmacology studies show that FF has a long duration of action and prolonged retention in the lung, suggesting it is appropriate for once-daily dosing [15,16]. As part of the overall FF clinical development program, a dose-ranging study (25–200 mcg doses of FF) showed that FF 50 mcg administered over 8 weeks was the minimum dose required to achieve significant improvements in evening trough forced expiratory volume in 1 s (FEV_1_) and the percentage of rescue-free 24-h periods compared with placebo [7].

This 12-week study sought to evaluate the efficacy and safety of once-daily FF 50 mcg dosed in the evening in asthma patients aged ≥12 years who were uncontrolled on short-acting β_2_-agonists (SABA) and/or leukotriene modifying agent. One other study with FF 50 mcg has been published [7], which was an 8-week dose ranging study. Two phase III studies of longer duration (of which this is one) comparing FF 50 mcg with placebo have been conducted in SABA only patients, to determine whether FF 50 mcg is a suitable starting dose for asthma patients not already using a controller medication. Preliminary results have been presented in abstract form [17].

## Methods

### Patients

Patients were aged ≥12 years with a diagnosis of asthma [4] made at least 12 weeks prior to screening and being treated with non-ICS controllers (a SABA alone or in combination with a leukotriene modifying agent); the use of ICS or LABA was not permitted for at least 4 weeks prior to the initial screening visit. Patients had to demonstrate a best FEV_1_ of ≥60% of the predicted normal value and ≥12% and 200 mL reversibility of FEV_1_ within 10–40 minutes following 2–4 inhalations of albuterol/salbutamol. Eligible patients also had no evidence of oral/oropharyngeal candidiasis.

At the end of a 2-week run-in period, patients were randomized if they had an evening pre-dose FEV_1_ ≥ 60% of the predicted normal value and, on at least 4 of the last 7 consecutive days of the run-in period, had documented use of albuterol/salbutamol and/or exhibited asthma symptoms and completed all morning and evening eDiary entries. Written informed consent was obtained from each patient.

### Study design and treatments

This was a phase III, multicentre, randomized, placebo-controlled, double-blind, parallel-group study conducted between 12th September 2011 and 7th August 2012 at 19 centers in four countries (Mexico, Peru, Russia, United States) (GSK study number FFA115283; www.clinicaltrials.gov registration number NCT01436071). The study was approved by local ethics committees and conducted in accordance with the Declaration of Helsinki and Good Clinical Practice guidelines.

Patients were randomized (1:1) to receive FF 50 mcg or placebo for a period of 12 weeks; both treatments were administered by the ELLIPTA dry powder inhaler once daily in the evening. Patients were randomized in accordance with a central randomization schedule generated by the sponsor using a validated computerized system (RandAll [GlaxoSmithKline, UK]), after a telephone call to the Registration and Medication Ordering System (RAMOS [GlaxoSmithKline, UK]). Both patients and investigators were blinded to treatment allocations. Treatment compliance was assessed by reviewing the dose counter on the ELLIPTA device. All patients received albuterol/salbutamol, to be used as needed throughout the run-in and treatment periods; no other asthma medications were permitted. The following non-asthma medications were permitted during the study: decongestants; intranasal and topical corticosteroids; immunotherapy; short- and long-acting antihistamines; and antihistamine eye drops.

### Outcome measurements

The primary efficacy endpoint was change from baseline in pre-dose evening (trough) FEV_1_ at Week 12. FEV_1_ measurements were performed in the clinic at Weeks 2, 4, 8 and 12 and were made within 1 h of the time FEV_1_ was measured at baseline and approximately 24-h after the last evening dose of medication.

The powered secondary endpoint was change from baseline in the percentage of rescue-free 24-h periods during the 12-week treatment period. Other secondary endpoints were: change from baseline in daily evening and morning peak expiratory force (PEF) averaged over the 12-week treatment period; change from baseline in the percentage of symptom-free 24-h periods during the 12-week treatment period; and number of withdrawals from study due to lack of efficacy. PEF measurements, symptoms and use of rescue medication were recorded daily using an eDiary.

Other selected endpoints included: change from baseline in Asthma Control Test^TM^ (ACT) score at Week 12; percentage of patients controlled (defined as having an ACT score ≥20) at Week 12; change from baseline in Asthma Quality of Life Questionnaire (AQLQ) + 12 Total score at Week 12; and ease of use questions on the ELLIPTA dry powder inhaler at the end of 4 weeks of treatment.

### Safety evaluations

Safety endpoints included the incidence of adverse events (AEs; coded using the Medical Dictionary for Regulatory Activities dictionary) and severe asthma exacerbations throughout the 12-week treatment period. A severe exacerbation was defined as deterioration of asthma requiring the use of systemic/oral corticosteroids for at least 3 days or an inpatient hospitalization or emergency department visit due to asthma that required systemic corticosteroids. Oropharyngeal examination was performed throughout the duration of the treatment period.

### Statistical analysis

A total of 220 randomized patients were expected to provide 104 evaluable patients per arm, giving 94% power to detect a difference of 200 mL between FF 50 mcg and placebo groups in evening trough FEV_1_, with significance declared at the two-sided 5% level. This also provided 95% power to detect a difference of 15% between FF 50 mcg and placebo groups in the change from baseline in percentage of rescue-free 24-h periods, with significance declared at the two-sided 5% level. The overall power of the study to detect treatment differences between FF 50 mcg and placebo for the primary and powered secondary endpoints was 90%.

The primary efficacy endpoint was the change from baseline in pre-dose evening (trough) FEV_1_ at the end of treatment (Week 12). This was analysed using an Analysis of Covariance (ANCOVA) model, which allowed for effects due to baseline FEV_1_, region, sex, age and treatment group. Last Observation Carried Forward was used to impute missing data. A supporting analysis was performed using a repeated measures model. Powered secondary, secondary and other endpoint comparisons were also analyzed using ANCOVA, with the exception of withdrawals due to lack of efficacy (analyzed using Fisher’s Exact test) and the percentage of patients controlled (ACT score ≥20; analyzed using logistic regression).

The safety population comprised all patients randomized to treatment and who received at least one dose of study medication. The intent-to-treat (ITT) population comprised all patients in the safety population except for 20 (10 from each treatment arm) patients excluded due to data quality issues identified through a site audit during a previous study. The per protocol population comprised all ITT patients who did not have any full protocol deviations.

In order to account for multiplicity across the key endpoints, a step-down closed testing procedure was applied for the primary and secondary endpoints whereby failure to achieve significance (*p* < 0.05) for the primary treatment comparison (FF 50 mcg vs. placebo), at any point in the hierarchy, meant that all tests lower down in the hierarchy were interpreted as descriptive only. The hierarchy was as follows: (1) evening trough FEV_1_; (2) rescue-free 24-h periods; (3) evening PEF; (4) morning PEF; (5) symptom-free 24-h periods; and (6) withdrawals due to lack of efficacy. If significance was achieved at each stage of the hierarchy, then all other efficacy endpoints were tested without further multiplicity adjustment.

## Results

### Study population

**Figure 1:**
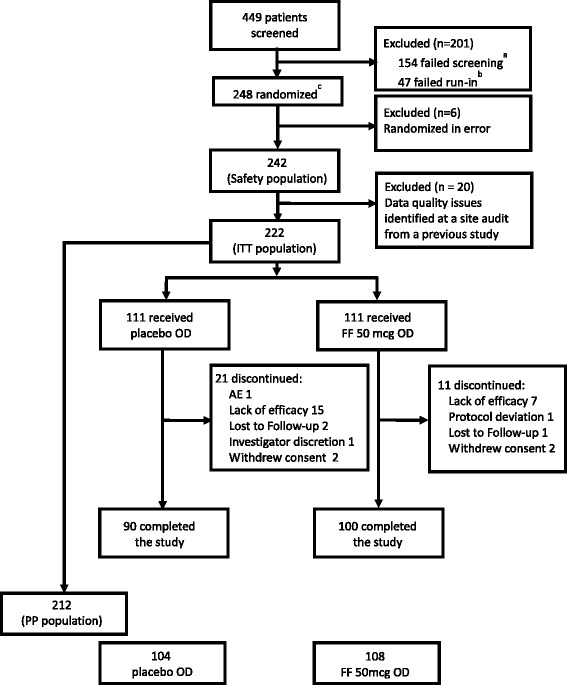
**Patient disposition.** ^a^Main reason was patients did not meet inclusion/exclusion criteria: 151(34%); ^b^main reason was patients did not meet continuation criteria: 36(8%); AE = adverse event; FF = fluticasone furoate; ITT = intent-to-treat; OD = once daily; PP = per protocol.

Of 449 patients screened, 248 were randomized, of whom 242 comprised the safety population and 222 comprised the ITT population. Within the ITT population, 90 (81%) placebo and 100 (90%) FF 50 mcg patients completed the study (Figure 1). Mean age, percentage of female patients and screening/baseline characteristics of lung function were similar between treatment groups (Table 1), with a baseline mean% predicted FEV_1_ of 81.58% in the ITT population. The majority of patients were of Hispanic/Latino ethnicity (73% placebo; 64% FF 50 mcg). Overall compliance with study medication (measured during the study using the dose counter on the DPI) was high and similar between treatment groups, at 97.7% in the placebo group and 99.0% in the FF 50 mcg group.

**Table 1:** **Patient demographics and lung function at screening/baseline (intent-to-treat population)**

	**Placebo OD PM (N = 111)**	**FF 50 mcg OD PM (N = 111)**
Age, mean (SD)	33.8 (13.90)	36.7 (16.16)
Age range, years	12–68	12–77
Female, n (%)	70 (63)	63 (57)
Race, n (%)		
American Indian or Alaska Native	59 (53)	45 (41)
White	29 (26)	42 (38)
American Indian or Alaska Native and White	21 (19)	24 (22)
Other	2 (2)	0
Screening characteristics, mean (SD)		
Pre-bronchodilator FEV_1_ (L)	2.527 (0.6940)	2.452 (0.6843)
Percent predicted FEV_1_, %	77.33 (12.884)	74.71 (9.493)
Post-bronchodilator FEV_1_ (L)	3.139 (0.8784)	3.047 (0.8443)
Percent reversibility FEV_1_, %	24.32 (10.368)	24.77 (9.906)
Baseline characteristics, mean (SD)		
Pre-bronchodilator FEV_1_* (L)	2.712 (0.8305)	2.669 (0.8172)
Percent predicted FEV_1_*, %	82.30 (14.115)	80.85 (12.277)
Rescue-free 24-h periods, %	7.5 (21.04)	10.2 (21.49)
Symptom-free 24-h periods, %	3.2 (12.73)	5.0 (14.53)
PM PEF (L/min)	356.8 (120.53)	359.0 (121.14)
AM PEF (L/min)	350.3 (115.89)	349.2 (117.21)

*Assessed in 110 patients from each treatment arm; FEV_1_ = forced expiratory volume in one second; FF = fluticasone furoate; OD = once daily; PEF = peak expiratory flow PM = evening; SD = standard deviation.

### Efficacy

**Figure 2:**
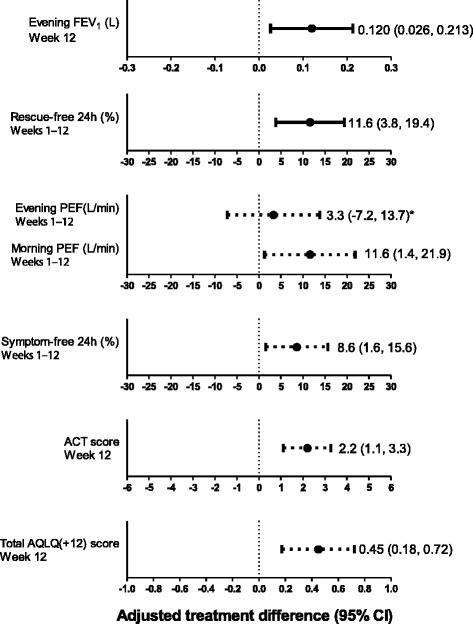
**Adjusted treatment differences for the change from baseline for primary, and selected secondary and other endpoints (intent-to-treat population).** *Data were analysed using a closed step-down statistical hierarchy, whereby failure to achieve significance at any point in the hierarchy meant that statistical significance could not be inferred for subsequent endpoints in the hierarchy. Solid lines indicate a significant treatment difference; dashed lines indicate differences which are not significant (evening PEF) or for which significance cannot be inferred (all remaining endpoints); FEV_1_ = forced expiratory volume in one second; PEF = peak expiratory flow; ACT = Asthma Control Test; AQLQ = Asthma Quality of Life Questionnaire; CI = confidence interval.

**Figure 3:**
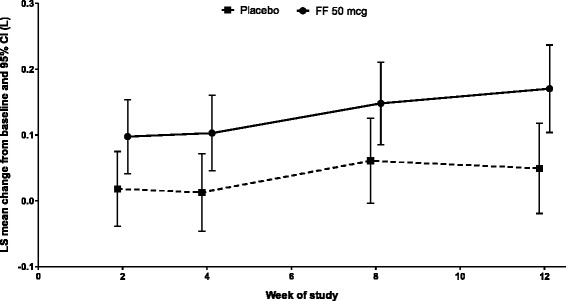
**Repeated measures analysis of change from baseline in trough FEV**_**1**_ **(L) (intent-to-treat population).** FF = fluticasone furoate; OD = once daily; LS = least squares; CI = confidence interval.

For the primary endpoint, the change from baseline evening trough FEV_1_ at Week 12 was 157 mL with FF 50 mcg and 38 mL with placebo; the treatment difference of 120 mL (*p =* 0.012) was statistically significant (Table 2; Figure 2). Repeated measures analysis demonstrated that trough FEV_1_ was consistently greater with FF 50 mcg vs. placebo throughout the study period (Figure 3). Supporting analysis of the per protocol population was similar (treatment difference in favor of FF 50 mcg: 131 mL [95% CI: 38, 224]; *p =* 0.006).

**Table 2:** **Results for primary, secondary and selected other endpoints (intent-to-treat population)**

	**Endpoint**	**Placebo OD PM (N = 111)**		**FF 50 mcg OD PM (N = 111)**		
		**n**	**Mean (SE)**	**n**	**Mean (SE)**	**Difference (95% CI)**
Pre-dose evening FEV_1_^a^, L	Primary	106	0.038 (0.0333)	108	0.157 (0.0330)	0.120* (0.026, 0.213)
Rescue-free 24-h periods^b^, %	Secondary (powered)	110	17.1 (2.78)	111	28.7 (2.77)	11.6*(3.8, 19.4)
Evening PEF^b^, L/min	Secondary	110	19.5 (3.72)	111	22.8 (3.70)	3.3 (−7.2, 13.7)
Morning PEF^b^, L/min	Secondary	110	22.9 (3.65)	111	34.5 (3.64)	11.6 (1.4, 21.9)
Symptom-free 24-h periods^b^, %	Secondary	110	14.0 (2.49)	111	22.6 (2.47)	8.6 (1.6, 15.6)
			**Number of patients**		**Number of patients**	
Withdrawal due to lack of efficacy^b^, %	Secondary		14		6	
		**n**	**Mean (SE)**	**n**	**Mean (SE)**	**Difference (95% CI)**
Asthma Control Test™ (ACT) score^a^	Other	92	4.0 (0.39)	100	6.2 (0.38)	2.2 (1.1, 3.3)
		**n**	**Number of patients**	**n**	**Number of patients**	**Odds Ratio (95% CI)**
Patients with ACT score ≥20^a^, %	Other	92	55	100	69	1.88 (0.97, 3.65)
		**n**	**Mean (SE)**	**n**	**Mean (SE)**	**Difference (95% CI)**
Total Asthma Quality of Life Questionnaire (AQLQ) + 12 score^a^	Other	92	0.84 (0.097)	100	1.30 (0.093)	0.45 (0.18, 0.72)

Change from baseline: ^a^at Week 12, ^b^during or averaged over Weeks 1–12. *statistically significant. FEV_1_ = forced expiratory volume in one second; PEF = peak expiratory flow; FF = fluticasone furoate; OD = once daily; PM = evening; SE = standard error; CI = confidence interval.

**Figure 4:**
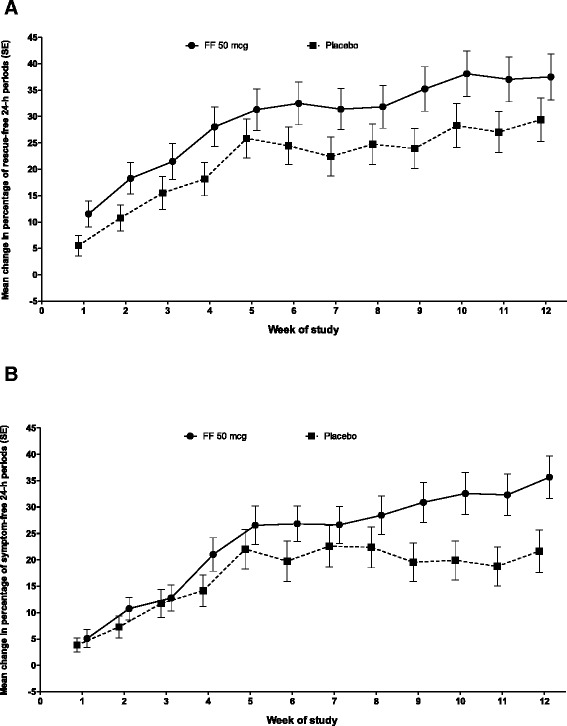
**Change from baseline in percentage of rescue-free (A) and symptom-free (B) 24-h periods during the 12-week study period (intent-to-treat population).** FF = fluticasone furoate; OD = once daily; SE = standard error.

For the powered secondary endpoint, there was a statistically significant increase from baseline in the percentage of rescue-free 24-h periods (28.7%) compared with placebo (17.1%; treatment difference of 11.6%; *p =* 0.004) (Table 2, Figure 2); this improvement equated to an additional 0.8 rescue-free 24-h periods per week with FF 50 mcg treatment. Rescue-free 24-h periods were consistently greater over the course of the study with FF 50 mcg compared with placebo (Figure 4A).

**Figure 5:**
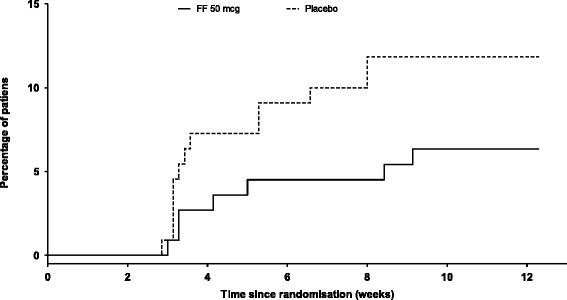
**Time to withdrawal due to lack of efficacy (intent-to-treat population).** FF = fluticasone furoate; OD = once daily.

The results for selected secondary and other endpoints are summarized in Table 2 and Figure 2. The change from baseline in evening PEF over the 12-week treatment period was increased with FF 50 mcg (22.8 L/min) and placebo (19.5 L/min), but the treatment difference (3.3 L/min) was not statistically significant (*p =* 0.536). In accordance with the pre-defined statistical hierarchy of endpoints, significance could not be inferred for the remaining endpoints. Increase from baseline in morning PEF was numerically greater for FF 50 mcg (34.5 L/min) compared with placebo treatment (22.9 L/min; treatment difference of 11.6 L/min). Likewise, the increase from baseline in the percentage of symptom-free 24-h periods was also numerically greater for FF 50 mcg (22.6%) compared with placebo treatment (14.0%; treatment difference of 8.6%), which equates to an additional 0.6 symptom-free 24-h periods per week with FF 50 mcg treatment. The greatest improvement in symptom-free 24-h periods with FF 50 mcg compared with placebo was observed during the last 4 weeks of the study (Figure 4B). A numerically greater proportion of patients in the placebo group withdrew due to lack of efficacy (14%) compared with patients in the FF 50 mcg group (6%) (Figure 5).

Numerically greater increases in ACT score, proportion of patients with an ACT score ≥20 and change from baseline in Total AQLQ(+12) score were observed for FF 50 mcg compared with placebo (Table 2; Figure 2). At baseline, most patients were able to use the ELLIPTA inhaler correctly after being instructed once (98% FF 50 mcg; 96% placebo). At Week 4, most patients rated the ELLIPTA inhaler as ‘easy/very easy’ to use (97%) and ‘easy/very easy’ to see how many doses of medication were left in the inhaler (95%).

### Safety assessments

Within the safety population, the overall incidence of on-treatment AEs was similar for placebo (38%) and FF 50 mcg (31%); the most frequently occurring on-treatment AEs were headache, nasopharyngitis, pharyngitis and influenza (Table 3). The incidence of treatment-related AEs was low in both treatment groups: 3% (n = 4) with placebo (pharyngitis, n = 1; upper respiratory tract infection, n = 1; dysgeusia and headache, n = 1; urticaria, n = 1); <1% (n = 1) with FF 50 mcg (contusion, n = 1). One patient in the placebo group was withdrawn from the study following an episode of urticaria. Additionally, three other AEs of special interest were reported: hypersensitivity (placebo), oropharyngeal pain (FF 50 mcg) and skeletal injury (FF 50 mcg). However, none of these were considered related to study medication or resulted in study withdrawal.

**Table 3:** **Summary of most frequent on-treatment AEs and serious AEs (safety population)**

**n, %**	**Placebo OD PM (N = 121)**	**FF 50 mcg OD PM (N = 121)**
**AEs**		
On treatment	46 (38)	37 (31)
On treatment, treatment-related	4 (3)	1 (<1)
On treatment, leading to withdrawal	1 (<1)^a^	0
Post treatment	1 (<1)	1 (<1)
**Serious AEs**		
On treatment^b^	1 (<1)	1 (<1)
**On treatment AEs occurring in ≥3% patients in either treatment group**		
Headache	14 (12)	6 (5)
Nasopharyngitis	5 (4)	7 (6)
Pharyngitis	6 (5)	6 (5)
Influenza	4 (3)	1 (<1)

^a^An incidence of urticaria in one placebo-treated patient; ^b^traffic accident, placebo group; perforated appendix, FF 50 mcg group; AE = adverse event; FF = fluticasone furoate; OD = once daily; PM = evening.

Two serious AEs were reported (traffic accident, placebo; perforated appendix, FF 50 mcg); neither was fatal nor considered to be treatment related, and both patients completed the study. Three patients in the placebo group experienced severe asthma exacerbations, while no patient in the FF 50 mcg group reported or was treated for a severe asthma exacerbation. There were no reports of pneumonia or oral/oropharyngeal candidiasis.

## Discussion

In patients with persistent asthma uncontrolled by non-ICS medications, FF 50 mcg administered once daily in the evening for 12 weeks significantly improved evening trough FEV_1_ compared with placebo. Patients who received FF 50 mcg also showed a significant increase in the percentage of rescue-free 24-h periods compared with placebo. The safety profile for FF 50 mcg was acceptable and similar to that of placebo. The patient population was chosen as the most appropriate for once-daily treatment with a low dose of FF.

The statistically significant improvement in evening trough FEV_1_ observed with FF 50 mcg compared with placebo (120 mL) is similar to findings from a recent dose-ranging study that investigated the potential of low doses of once-daily FF (25–200 mcg) for the treatment of persistent asthma over 8 weeks [7]. The current study was performed in a similar population of asthma patients (i.e., those who required a step-up to Step 2 of asthma treatment guidelines) and, although lung function at baseline was different, those authors found that FF 50–200 mcg statistically significantly improved lung function compared with placebo; the treatment difference between FF 50 mcg and placebo in trough FEV_1_ was 129 mL (*p* < 0.033). The present data for FF, generated from a larger cohort of patients and over a longer period of time, support these findings [7]. The study was powered based on a change in evening trough FEV_1_ of 200 mL. However, in another study comparing morning and evening dosing of FF/VI whose findings were reported after our study design was finalized, smaller treatment differences vs. placebo were seen for evening FEV_1_ when compared with morning FEV_1_ – this was probably due to the known diurnal variation in lung function [18]. Despite this, the effect of FF 50 mcg on evening FEV_1_ in our study was significant. However, in another phase III study the improvement for FF 50 mcg over placebo was not statistically significant, although improvement with the active comparator fluticasone propionate (FP) 100 mcg was significant (102 mL; *p =* 0.030) [14]. The reason for the findings of Busse et al. not being consistent with those of the current and previous studies of FF 50 mcg [7] is unclear, as the patient population enrolled was very similar to that of the current study. Finally, for the powered secondary endpoint in this study, the statistically significant increase in the percentage of rescue-free 24-h periods with FF 50 mcg compared with placebo is consistent with results from the 8-week dose-ranging study that involved FF 50 mcg [7]. Collectively, the findings suggest that FF 50 mcg results in meaningful improvements in trough FEV_1_ and percentage of rescue-free 24-h periods.

Statistical significance was not achieved for the secondary endpoint of change from baseline in evening PEF for FF 50 mcg vs. placebo, meaning that significance could not be inferred for the remaining secondary and other endpoints. However, other studies investigating treatment with FF 50 mcg [7,14] or FF 100 mcg [8] have reported numerically greater improvements in evening PEF compared with placebo (treatment differences of 20.7 L/min, 17.2 L/min and 15.9 L/min, respectively). Lung function is generally improved in the evening due to diurnal variation [19], reducing the likelihood of detecting treatment benefit at that time point. However, trough FEV_1_, which was also assessed in the evening, did improve significantly following FF 50 mcg compared with placebo treatment. For the remaining endpoints of morning PEF, symptom-free 24-h periods, ACT score, percentage of patients controlled (defined by ACT score ≥20 at Week 12) and Total AQLQ score, numerically greater increases were observed over 12 weeks in patients who received FF 50 mcg compared with placebo. More patients in the placebo group were withdrawn due to lack of efficacy compared with the FF 50 mcg group.

The overall safety profile was favorable in both the FF 50 mcg and placebo groups, consistent with previous findings for this dose of FF [7,14]. Cortisol levels were not measured in this study, as the treatment was with a low dose of FF and no effect of cortisol had been seen with higher doses. The effect of FF on cortisol levels has been assessed in a separate meta-analysis, which is published elsewhere [20]. Common AEs experienced by asthma patients receiving ICS treatment included headache, nasopharyngitis, pharyngitis and influenza. Incidence of these AEs was similarly low between treatment groups in this study, although headache was more frequent in the placebo group.

A strength of our study is the inclusion of a statistical hierarchy of endpoints, which added robustness in validating the overall efficacy of FF 50 mcg compared with placebo. However, this might also be considered a weakness, as failure to achieve statistical significance for evening PEF meant that treatment differences between FF 50 mcg and placebo for the remaining endpoints could only be interpreted in descriptive terms. Another strength was the use of electronic diary cards, which meant that all entries were date and time stamped and which did not allow retrospective entries; this is likely to have increased the quality of daily recordings.

## Conclusions

In summary, FF 50 mcg administered once daily in the evening significantly improved evening trough FEV_1_ and the percentage of rescue-free 24-h periods compared with placebo. Improvements with FF 50 mcg were numerically greater than with placebo for all efficacy endpoints. Fewer patients receiving FF 50 mcg treatment withdrew due to lack of efficacy compared with patients receiving placebo treatment and there were no safety concerns. Overall, the efficacy/tolerability profile of FF 50 mcg was acceptable, suggesting that treatment with FF 50 mcg is potentially suitable for patients ≥12 years of age with persistent asthma that is uncontrolled by non-ICS therapy.

### Endnote

^a^ELLIPTA™ is a trademark of the GlaxoSmithKline group of companies.

## Abbreviations

ACT: Asthma control test™
AE: adverse events
ANCOVA: Analysis of covariance
AQLQ: Asthma quality of life questionnaire
FEV_1_: forced expiratory volume in one second
FF: Fluticasone furoate
ICS: Inhaled corticosteroid
ITT: Intent-to-treat
LABA: Long-acting β_2_-agonist
PEF: Peak expiratory force
SABA: Short-acting β2-agonists
VI: Vilanterol
